# Telemedicine and Plastic Surgery in India

**Published:** 2014-01

**Authors:** Nikhil Panse

**Affiliations:** Department of Plastic Surgery, BJ Medical College and Sassoon Hospital, Pune, India

**Dear Editor**

I read with great interest the article of ‘Telemedicine in Iran: Chances and challenges’ while the article reviewed the evolution of telemedicine and studied the perception of telemedicine in the medical fraternity in Iran.^1^ Even though the concept of telemedicine is old, it has recently started to gain widespread publicity within the field of plastic surgery, where visual examination contributes heavily to patient management and decision-making.^2^

In a country like India, where there are a handful of plastic surgeons as compared to the overall population, the utility of telemedicine seems immense. We have started using telemedicine in the recent times for the benefit of our patients too. I work in a tertiary care government institute. In our state, there is a network of telemedicine centers which are attached to our tertiary care centre with facilities for exchange of clinical information. 

Whenever a request for a teleconsultation is received from one of the centers for plastic surgery, our consultant does the necessary consultation, investigations are recommended as and when necessary, and a follow up consultation if needed is also provided. If the patient needs surgical intervention, he/she is advised a date for admission to our tertiary care centre. If there is no need for surgical intervention, the patient is counseled otherwise. This method has many advantages, especially in the government set up as follows:

(i). The patient gets a direct date for admission, reducing the multiple visits to the hospital, and (ii). In those cases like clefts, where surgery is not considered because of less age/or low hemoglobin, it gives us an opportunity to counsel the patients regarding dietary/feeding advise and regular follow up.

In a country like India, where many patients turning out to government institutes are daily wage workers, telemedicine is an affordable and effective way to reduce the expenses and travel time. Many times, elective surgeries have a waiting period of up to three months in a tertiary care government institute like ours. Telemedicine helps us in giving a date accordingly to the patient, so that the patient can directly come for admission without any prior visits.

Many patients are lost to follow up simply because they cannot afford to come repeatedly for long distances. This results in a lot of clinical data remaining incomplete without long term follow up. Telemedicine centers located across the state are nearer to the patients, thereby giving us an opportunity to interact with patients and take clinical photographs. We have also been using this facility for teleeducation. 

Plastic surgery is a poorly understood branch of medicine, especially by our medical colleagues.^3^ Through teleeducation, we try to educate the medical and paramedical staff as to the scope and extent of plastic surgery, thereby increasing the number of direct referrals to our specialty. Our use and application of telemedicine in plastic surgery is still in its infancy, and our spectrum is limited to elective cases. But our early experience suggests immense potential in its application especially for government institutes for the benefit of our patients.

Although separated geographically by a few countries and some mountain ranges and a few kilometers, Iran and India actually have many things in common. Inadequate number of tertiary healthcare facilities and centers as compared to the population being one of them. Telemedicine can be an important tool in the hands of the plastic surgical fraternity to manage our patients in a more effective manner in and across the two countries. 

However, there is little critical analysis on the benefits and risks of telemedicine. Detailed long term evaluation and its legal implications need to be carefully considered if it is to be safely incorporated into our daily practice.

## CONFLICT OF INTEREST

The authors declare no conflict of interest.

## Footenote

Please cite this paper as:

Panse N. Telemedicine and Plastic Surgery in India. World J Plast Surg 2014;3(1):70-71.
